# Depression and Anxiety Among Obese and Overweight Individuals in Saudi Arabia: A Systematic Review and Meta-Analysis

**DOI:** 10.7759/cureus.85907

**Published:** 2025-06-13

**Authors:** Amer H Alshahre, Saad A Alqahtani, Maryam S Alsharif, Alaa A Alyahya, Maher A Alsmail, Abdulaziz S Almasabi, Waleed S Abumelha

**Affiliations:** 1 Family Medicine, Armed Forces Hospital Southern Region, Khamis Mushait, SAU; 2 Psychiatry, Armed Forces Hospital Southern Region, Khamis Mushait, SAU; 3 Family Medicine, Alsharqia Primary Health Care Center, Ministry of Health, Yanbu, SAU; 4 Community Medicine, King Khalid University, Abha, SAU; 5 Family Medicine, Eastern Cluster of Ministry of Health, Dammam, SAU; 6 Family Medicine, Riyadh Second Cluster, Ministry of Health, Riyadh, SAU; 7 Obesity and Eating Disorders, Armed Forces Hospital Southern Region, Khamis Mushait, SAU

**Keywords:** anxiety, depression, meta-analysis, obesity, saudi arabia

## Abstract

The escalating prevalence of obesity in Saudi Arabia raises concerns about its impact on mental health. This meta-analysis explores the associations between obesity and the prevalence of depression and anxiety within the distinctive cultural context of Saudi Arabia. A systematic review identified 12 cross-sectional studies conducted in Saudi Arabia, collectively involving 17,232 participants. Pooled effect sizes were calculated for depression and anxiety outcomes among obese and overweight individuals. Heterogeneity was assessed, and subgroup analyses were planned to explore potential sources of variation. The meta-analysis revealed a significant association between obesity and overall depression (OR = 1.42, 95% CI (1.07, 1.87)), severe depression (OR = 1.76, 95% CI (1.08, 2.86)), and severe anxiety (OR = 1.62, 95% CI (1.24, 2.11)). Severe anxiety was also significantly associated with overweight status (OR = 1.46, 95% CI (1.12, 1.90)). However, no significant associations were found between depression and overweight status. Subgroup analyses were hindered by limited data. The overall prevalence of depression among obese individuals was 25.02%, and for anxiety, it was 29.23%. This meta-analysis provides robust evidence supporting the association between obesity and both severe depression and severe anxiety in the Saudi Arabian population. The findings underscore the importance of addressing mental health issues in individuals with higher BMIs. Future research should explore the underlying mechanisms of this association and evaluate the effectiveness of tailored interventions to promote holistic well-being in this population. These insights contribute to the development of targeted public health strategies for addressing the complex interplay between mental health and weight status in Saudi Arabia.

## Introduction and background

The Kingdom of Saudi Arabia has witnessed a remarkable epidemiological transition over the past few decades, marked by a surge in noncommunicable diseases [1,2]. Among these, obesity stands out as a pressing public health concern, with far-reaching implications for both physical and mental well-being [3]. As the prevalence of obesity continues to rise globally, the complex interplay between excess body weight and mental health outcomes has garnered increased attention [4].

Saudi Arabia, like many countries worldwide, has experienced a significant increase in the prevalence of obesity [5]. Rapid urbanization, changes in dietary patterns, and sedentary lifestyles have contributed to the escalating rates of overweight and obesity in the population [6]. According to national surveys, the prevalence of obesity among adults in Saudi Arabia has surged from 22.4% in 1995 to 35.4% in 2017, underscoring the urgency of addressing this burgeoning epidemic [4-7].

The consequences of obesity extend beyond physical health, encompassing a spectrum of mental health outcomes [8]. The intricate relationships between obesity, depression, and anxiety have been a subject of extensive research globally [8-12]. However, understanding these connections within the specific cultural and contextual nuances of Saudi Arabia is vital for tailoring effective public health interventions.

Mounting evidence suggests bidirectional associations between obesity and mental health conditions. Individuals with obesity may experience stigma, discrimination, and societal pressure, contributing to psychosocial distress [13,14]. Concurrently, mental health challenges may influence behaviors related to diet and physical activity, exacerbating the risk of obesity. This reciprocal relationship creates a complex web that warrants careful examination [15].

Depression and anxiety, two prevalent mental health disorders, have been consistently linked to obesity in diverse populations [16]. The psychosocial impact of obesity, coupled with potential physiological mechanisms involving inflammation and hormonal dysregulation, forms the basis for exploring these associations [17]. Understanding the nuanced nature of these relationships is crucial for informing comprehensive healthcare strategies that address the dual burden of obesity and mental health issues [18,19].

While individual studies have explored the connections between obesity, depression, and anxiety in Saudi Arabia, synthesizing the existing evidence through a meta-analysis provides the highest level of evidence-based medicine. This approach allows for the quantification of associations, the identification of patterns across diverse populations, and the exploration of potential sources of heterogeneity. Moreover, a meta-analysis enables the examination of the collective impact of obesity on varying degrees of depression and anxiety, shedding light on the nuances of these relationships. This meta-analysis aims to explore the associations between obesity and two prevalent mental health conditions, depression and anxiety, within the sociocultural context of Saudi Arabia, which will subsequently contribute to the general literature on this subject.

## Review

Materials and methods

This study is a meta-analysis that adhered to the Preferred Reporting Items for Systematic reviews and Meta-Analyses (PRISMA) guidelines, ensuring transparency and completeness in reporting the study methodology and results. The PRISMA checklist was systematically followed to enhance the rigor and reproducibility of the meta-analysis [20]. The methodology commenced with an exhaustive literature search conducted across electronic databases, including PubMed, Scopus, and PsycINFO, covering articles published up to the cutoff date of January 2022. The search strategy utilized a combination of controlled vocabulary (MeSH terms) and free-text keywords related to depression, anxiety, obesity, overweight, and Saudi Arabia. Boolean operators (AND, OR) were employed to refine the search and ensure inclusivity.

Study Search and Screening Process

Inclusion criteria were systematically applied at each stage of the screening process. Duplicates were identified and removed. Title and abstract screening followed, with records undergoing evaluation based on predetermined eligibility criteria. Studies were included if they were cross-sectional in design, conducted in Saudi Arabia, focused on individuals with obesity or overweight status, and reported quantitative data on the prevalence of depression and anxiety. Subsequently, studies were sought for retrieval, and the remaining studies were meticulously assessed for eligibility by two independent reviewers. Studies that did not meet the inclusion criteria or lacked relevant outcome measures were excluded.

Data Extraction

A standardized data extraction form was developed to systematically extract relevant information from the included studies. Two independent reviewers extracted data, including study characteristics (e.g., author and publication year), study design, setting, city, study duration, population type, population number, age distribution, sex distribution, BMI trends, diagnostic tools for depression and anxiety, and overall rates of depression and anxiety. For each outcome of interest (depression and anxiety), data on prevalence, ORs, and associated 95% CIs were extracted. Special attention was given to data related to different levels of depression and anxiety, such as moderate and severe categories, when reported by the included studies.

Statistical Analysis

The statistical synthesis involved the use of the Review Manager software, version 5.4. Pooled effect sizes (ORs) were calculated for each outcome of interest using a random-effects model to account for potential heterogeneity among the included studies. The I² statistic was used to assess heterogeneity. Forest plots were generated to visually represent the effect sizes and associated CIs for each outcome. Subgroup analyses were planned to explore potential sources of heterogeneity. Additionally, publication bias was evaluated using funnel plots and Egger’s regression test.

Results

**Figure 1:**
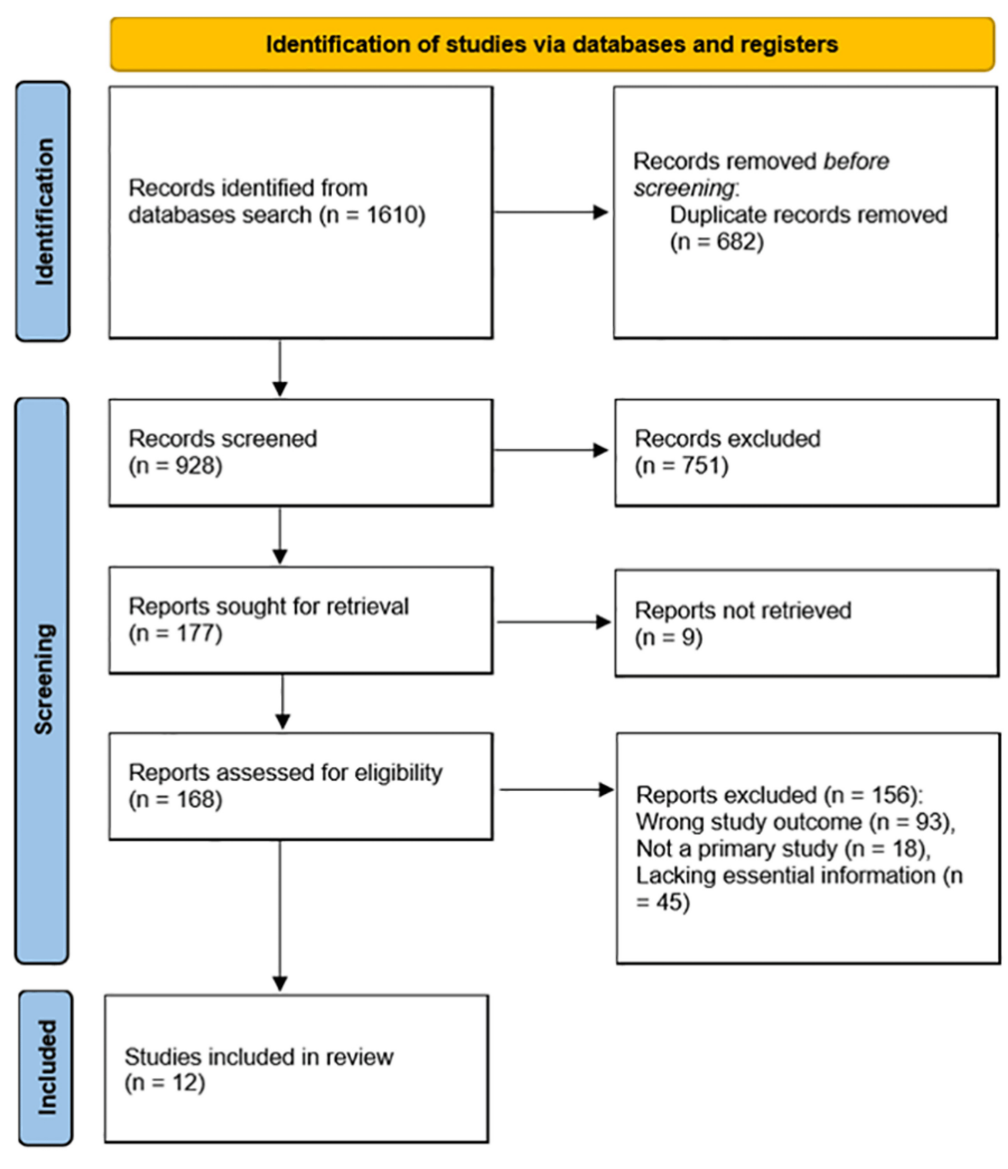
PRISMA flow diagram for summary of the study selection process PRISMA, Preferred Reporting Items for Systematic reviews and Meta-Analyses

A systematic review of the literature was conducted, yielding 1610 records, from which 682 duplicates were removed. After title and abstract screening, 928 records were enrolled, leading to the exclusion of 751 studies. Subsequently, 177 records were retrieved, with nine studies not obtained. A total of 168 studies were assessed for eligibility, and 156 were excluded, resulting in 12 studies being included in the meta-analysis (Figure 1).

Characteristics of the Included Studies

Table 1 provides a summary of the included studies.

**Table 1:** Characters of the included studies (n = 12) BDI-II, Beck Depression Inventory-II; DASS, Depression Anxiety Stress Scale; GAD-7, Generalized Anxiety Disorder-7; HAD-A, Hospital Anxiety and Depression Scale - Anxiety Subscale; HAD-D, Hospital Anxiety and Depression Scale - Depression Subscale; NA, not applicable; NR, not reported; PHCC, primary health care center; PHQ-9, Patient Health Questionnaire-9; PQ-2, Patient Health Questionnaire-2

Study	Study design	Study setting	City	Study duration	Population type	Population number	Age (year)	Males (%)	Normal BMI (%)	Overweight (%)	Obese (%)	Diagnostic tool for depression	Diagnostic tool for anxiety	Overall depression rate among the entire population (%)	Overall anxiety rate among the entire population (%)
Abbas et al. (2012) [21]	Cross-sectional study	Hospital-based	Riyadh	2011	Nurses	715	35.2 ± 8.2	11.60%	56.50%	Overweight and obese: 38.46%		HAD-D	HAD-A	10%	20%
Alhusseini et al. (2021) [22]	Cross-sectional study	Community-based	Nonspecific	2020	General public	1,123	18-29	29.11%	34.55%	31.79%	28.67%	NA	GAD-7	NA	36.33%
Aljurbua et al. (2021) [23]	Cross-sectional study	Community-based	Nonspecific	2021	General public in KSA	338	18-64	39.30%	36.30%	28%	28%	NA	GAD-7	NA	29%
Alkharji et al. (2023) [24]	Cross-sectional study	Hospital-based	Jeddah	2023	Adults visiting PHCCs	397	NR	56.90%	15.60%	17.60%	65.50%	PHQ-9	NA	29.70%	NA
Alkot et al. (2019) [25]	Cross-sectional study	Community-based	Makkah	2018-2019	Diabetic population	169	40.27 + 15.95	69.20%	NR	NR	30.20%	BDI-II	NA	24.30%	NA
Almarhapi and Khalil (2021) [26]	Cross-sectional study	Hospital-based	Tabuk	2021	Healthcare workers	255	30.6 ± 5.3	41.20%	64.70%	23.90%	7.10%	PHQ-9	NA	11.76%	NA
Almarhoon et al. (2021) [27]	Cross-sectional study	Community-based	Eastern Province	2020-2021	Saudi adults	711	18-65	24.90%	35.70%	30.50%	26.40%	PHQ-9	NA	34.80%	NA
AlQahtani et al. (2015) [28]	Cross-sectional study	Community-based	Abha	2013-2014	Male university students	389	21.2 ± 1.5	100%	46.20%	22.10%	18.30%	DASS	DASS	29.30%	48.30%
Baeisa et al. (2023) [29]	Cross-sectional study	Community-based	Nonspecific	2021-2023	General public	4,224	31.12 ± 11	39.10%	42%	27.96%	19.53%	NA	GAD-7	NA	29%
Darwish et al. (2014) [30]	Cross-sectional study	Hospital-based	Qatif	2013-2014	Public attending PHCCs	630	34.3 ± 9.3	0%	34.60%	29%	36%	DASS	DASS	21.40%	29.20%
Joury (2014) [31]	Cross-sectional study	Community-based	Riyadh	2012	General public	787	NR	69.80%	24%	33.16%	40.15%	BDI-II	NA	17.40%	NA
Sultan et al. (2016) [32]	Cross-sectional study	Community-based	Madina	2014	Medical students	555	NR	47%	56.75%	22.16%	12.07%	PQ-2	NA	28.28%	NA

All studies adopted a cross-sectional study design. The settings varied, including hospital-based studies such as Abbas et al. (2012) [21], Alkharji et al. (2023) [24], Almarhapi and Khalil (2021) [26], and Darwish et al. (2014) [30]. Community-based studies were conducted by Alhusseini et al. (2021) [22], Aljurbua et al. (2021) [23], Alkot et al. (2019) [25], Almarhoon et al. (2021) [27], AlQahtani et al. (2015) [28], Baeisa et al. (2023) [29], Joury (2014) [31], and Sultan et al. (2016) [32]. The studies were conducted in various cities, including Riyadh, Jeddah, Makkah, Tabuk, Eastern Province, Abha, and Qatif. The durations of the studies ranged from 2011 to 2023, with a focus on different time periods.

The populations under investigation were diverse, encompassing nurses [21], the general public [22,23,26,27,29,31], adults visiting primary health care centers [24], diabetic population [25], healthcare workers [26], male university students [28], and medical students [32]. The population numbers ranged from 169 to 4,224.

Age distribution varied across studies, with age ranges specified for each study. Males constituted different percentages, ranging from 0% [30] to 100% [28]. BMI trends showed variability, with studies reporting the percentage of normal weight, overweight, and obese individuals.

Studies utilized various tools for assessing depression and anxiety. The Hospital Anxiety and Depression Scale (HAD-D and HAD-A) was used in Abbas et al. (2012) [21], and the Depression, Anxiety, and Stress Scale (DASS) in AlQahtani et al. (2015) [28] and Darwish et al. (2014) [30]. The Patient Health Questionnaire-9 (PHQ-9) was employed by Alkharji et al. (2023) [24] and Almarhoon et al. (2021) [27], and the Beck Depression Inventory-II (BDI-II) in Alkot et al. (2019) [25] and Joury et al. (2014) [31]. The Generalized Anxiety Disorder 7-item scale (GAD-7) was used in Alhusseini et al. (2021) [22], Aljurbua et al. (2021) [23], Almarhapi and Khalil (2021) [26], and Baeisa et al. (2023) [29]. Sultan et al. (2016) [32] used the Patient Health Questionnaire-2 (PHQ-2).

Depression rates ranged from 10% to 34.8%, with the highest rate observed in Almarhoon et al. (2021) [27]. Anxiety rates ranged from 20% to 48.3%, with the highest rate reported in AlQahtani et al. (2015) [28].

Quantitative Data Synthesis

Table 2 presents the pooled effect sizes and heterogeneity assessment for the assessed outcomes, offering a comprehensive overview of the prevalence of depression and anxiety among both obese and overweight individuals in Saudi Arabia.

**Table 2:** Pooled effect sizes and heterogeneity assessment for the assessed outcomes Chi², chi-square test for heterogeneity; df, degrees of freedom; FE, fixed-effects model; I², percentage of total variation across studies due to heterogeneity; RE, random-effects model; Tau², between-study variance; Z, Z test statistic for overall effect

Measure	Number of studies	Participants (N)	Prevalence (%)	Heterogeneity	Model	OR	Test for overall effect
Depression among obese individuals							
Moderate and severe depression	9	3,344	25.02%	Tau² = 0.09; Chi² = 17.33, df = 8 (P = 0.03); I² = 54%	RE	1.42 (1.07, 1.87)	Z = 2.47 (P = 0.01)
Moderate depression	4	1,351	15.03%	Tau² = 0.25; Chi² = 9.52, df = 3 (P = 0.02); I² = 68%	RE	1.42 (0.78, 2.60])	Z = 1.14 (P = 0.25)
Severe depression	4	1,351	7.01%	Chi² = 2.88, df = 3 (P = 0.41); I² = 0%	FE	1.76 (1.08, 2.86)	Z = 2.28 (P = 0.02)
Depression among overweight individuals							
Moderate and severe depression	7	2,384	24.58%	Chi² = 4.86, df = 6 (P = 0.56); I² = 0%	FE	1.12 (0.92, 1.37)	Z = 1.16 (P = 0.25)
Moderate depression	4	1,343	14.04%	Chi² = 3.08, df = 3 (P = 0.38); I² = 2%	FE	1.04 (0.76, 1.44)	Z = 1.16 (P = 0.25)
Severe depression	4	1,343	5.92%	Chi² = 1.02, df = 3 (P = 0.80); I² = 0%	FE	1.46 (0.89, 2.39)	Z = 1.51 (P = 0.13)
Anxiety among obese individuals							
Moderate and severe anxiety	6	4,866	29.23%	Tau² = 0.18; Chi² = 31.00, df = 5 (P < 0.00001); I² = 84%	RE	1.28 (0.87, 1.86)	Z = 1.26 (P = 0.21)
Moderate anxiety	4	1,584	16.35%	Tau² = 0.19; Chi² = 8.98, df = 3 (P = 0.03); I² = 67%	RE	1.22 (0.72, 2.06)	Z = 0.74 (P = 0.46)
Severe anxiety	4	1,584	20.77%	Chi² = 2.86, df = 3 (P = 0.41); I² = 0%	FE	1.62 (1.24, 2.11)	Z = 3.56 (P = 0.0004)
Anxiety among overweight individuals							
Moderate and severe anxiety	5	4,582	29.07%	Tau² = 0.04; Chi² = 8.75, df = 4 (P = 0.07); I² = 54%	RE	1.04 (0.82, 1.32)	Z = 0.29 (P = 0.77)
Moderate anxiety	4	1,623	15.59%	Chi² = 1.03, df = 3 (P = 0.79); I² = 0%	FE	0.88 (0.67, 1.15)	Z = 0.94 (P = 0.35)
Severe anxiety	4	1,623	19.49%	Chi² = 1.03, df = 3 (P = 0.79); I² = 0%	FE	1.46 (1.12, 1.90)	Z = 2.84 (P = 0.005)

Depression Among Obese Individuals

**Figure 2:**
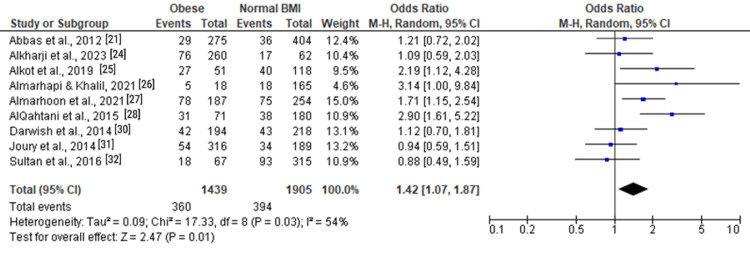
Forest plot of moderate and severe depression among obese versus normal BMI individuals

**Figure 3:**
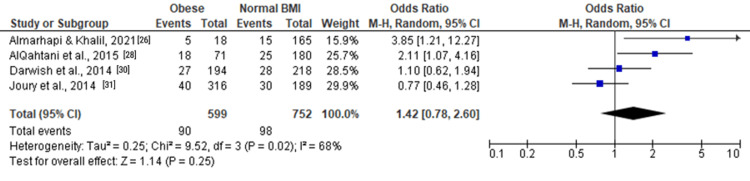
Forest plot of moderate depression among obese versus normal BMI individuals

**Figure 4:**
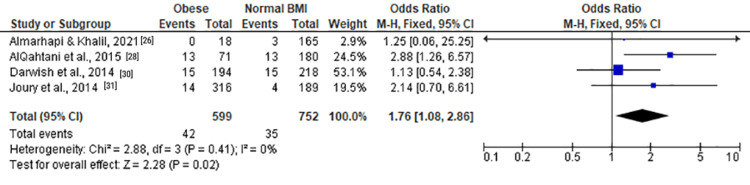
Forest plot of severe depression among obese versus normal BMI individuals

For moderate and severe depression (Figure 2), the pooled OR was 1.42 (1.07, 1.87), indicating a significant association with obesity (Z = 2.47, P = 0.01). Moderate depression (Figure 3) showed a similar trend with an OR of 1.42 (0.78, 2.60), although not statistically significant (Z = 1.14, P = 0.25). Severe depression (Figure 4) demonstrated a significant association with obesity, with an OR of 1.76 (1.08, 2.86) (Z = 2.28, P = 0.02).

Depression Among Overweight Individuals

**Figure 5:**
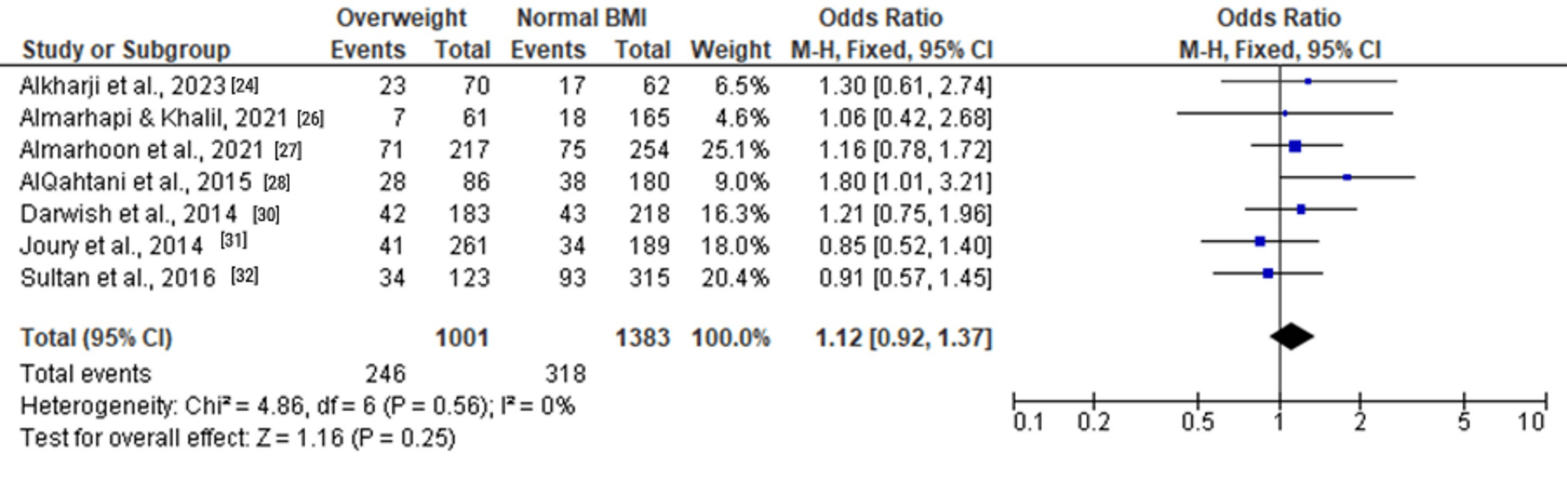
Forest plot of moderate and severe depression among overweight versus normal BMI individuals

**Figure 6:**
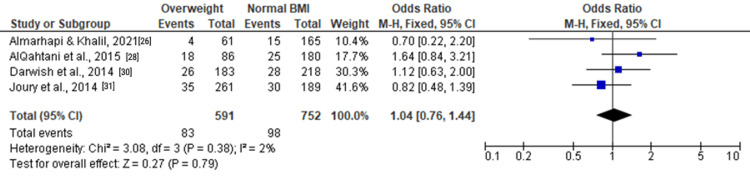
Forest plot of moderate depression among overweight versus normal BMI individuals

**Figure 7:**
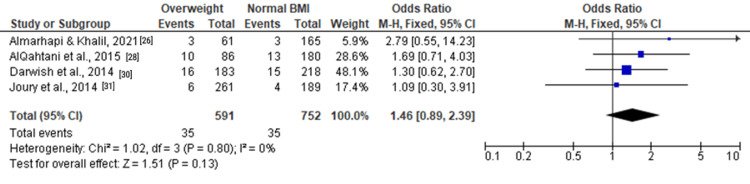
Forest plot of severe depression among overweight versus normal BMI individuals

Among overweight individuals, the forest plots in Figure 5, Figure 6, and Figure 7 depicted no statistically significant associations with depression. The ORs for moderate and severe depression were 1.12 (0.92, 1.37), 1.04 (0.76, 1.44), and 1.46 (0.89, 2.39), respectively.

Anxiety Among Obese Individuals

**Figure 8:**
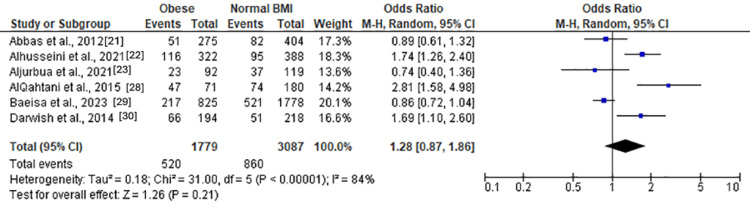
Forest plot of moderate and severe anxiety among obese versus normal BMI individuals

**Figure 9:**
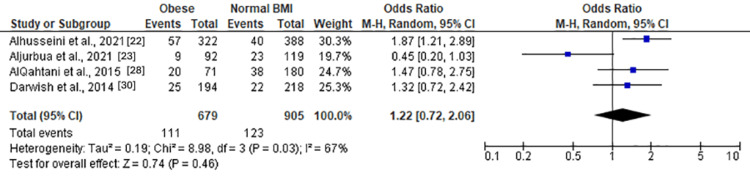
Forest plot of moderate anxiety among obese versus normal BMI individuals

**Figure 10:**
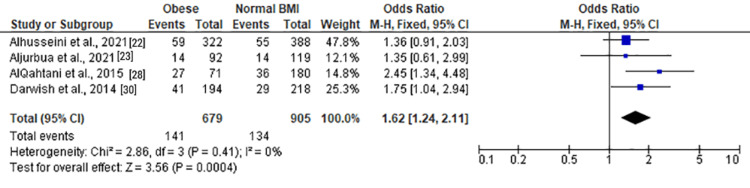
Forest plot of severe anxiety among obese versus normal BMI individuals

Moderate and severe anxiety (Figure 8) revealed a nonsignificant association with obesity (OR = 1.28 (0.87, 1.86), Z = 1.26, P = 0.21). Moderate anxiety (Figure 9) also exhibited a nonsignificant association (OR = 1.22 (0.72, 2.06), Z = 0.74, P = 0.46). However, severe anxiety (Figure 10) demonstrated a significant association with obesity, with an OR of 1.62 (1.24, 2.11) (Z = 3.56, P = 0.0004). Heterogeneity was absent (Chi² = 2.86, df = 3, P = 0.41; I² = 0%).

Anxiety Among Overweight Individuals

**Figure 11:**
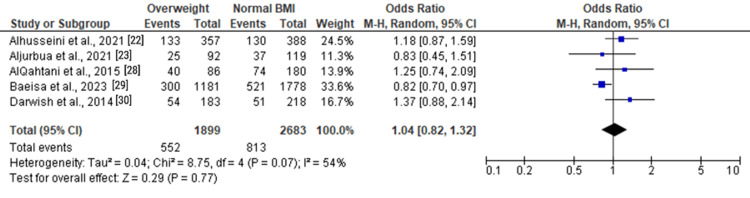
Forest plot of moderate and severe anxiety among overweight versus normal BMI individuals

**Figure 12:**
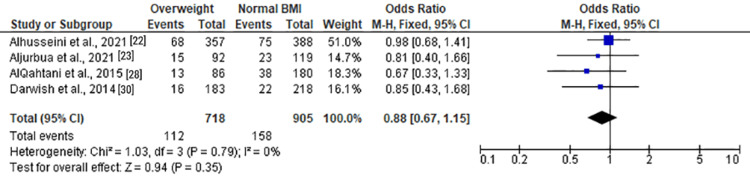
Forest plot of moderate anxiety among overweight versus normal BMI individuals

**Figure 13:**
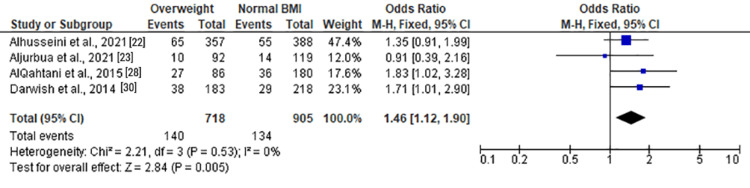
Forest plot of severe anxiety among overweight versus normal BMI individuals

Among overweight individuals, the forest plots in Figure 11 and Figure 12 illustrated no statistically significant associations with anxiety. The ORs for overall and moderate anxiety were 1.04 (0.82, 1.32) and 0.88 (0.67, 1.15), respectively. Severe anxiety, as shown in Figure 13, was significantly associated with overweight status with an OR of 1.46 (1.12, 1.90) (Z = 2.84, P = 0.005).

Discussion

Obesity is a global health concern with multifaceted implications, and its association with mental health, particularly depression and anxiety, has garnered significant research interest [33,34]. In the context of Saudi Arabia, where the prevalence of obesity is escalating, understanding the interplay between obesity and mental health is crucial [35]. This meta-analysis aimed to consolidate and analyze data from 12 cross-sectional studies to elucidate the prevalence of depression and anxiety among obese and overweight individuals in Saudi Arabia.

Our meta-analysis yielded compelling insights into the relationship between obesity and mental health in the Saudi Arabian population. In terms of depression among obese individuals, the pooled OR for overall depression was 1.42 (1.07, 1.87), indicating a significant association. Moreover, the observed OR for severe depression was 1.76 (1.08, 2.86), reinforcing the association between obesity and more severe forms of depressive symptoms [36,37]. These results coincide with the growing body of literature suggesting that obesity is a risk factor for the development and exacerbation of depression, as highlighted in studies such as AlQahtani et al. (2015) [28].

Contrastingly, the analysis of depression among overweight individuals did not reveal statistically significant associations. This nuanced difference in the association between depression and different weight statuses echoes the findings of Baeisa et al. (2023) [29], where depression rates were observed in the general public but did not show a significant correlation with overweight status.

As regards anxiety, our findings demonstrate that among obese individuals, severe anxiety exhibited a significant association with an OR of 1.62 (1.24, 2.11). However, the associations with moderate anxiety and moderate and severe anxiety combined did not reach statistical significance. This mirrors the complexity of the relationship between obesity and anxiety [38], as suggested by Alhusseini et al. (2021) [22], who found a nonsignificant association in a community-based study.

Similarly, among overweight individuals, our analysis did not unveil statistically significant associations with overall or moderate anxiety. These results are in line with the findings of Joury et al. (2014) [31], where anxiety rates in the general public in Riyadh did not exhibit a significant correlation with overweight status. However, the study found a significant association between overweight state and severe anxiety [39].

The observed significant association between obesity and both severe depression and severe anxiety underscores the need for comprehensive mental health interventions among individuals with higher BMIs [37,39,40]. Our findings align with previous meta-analyses and systematic reviews that have identified obesity as a risk factor for the development and exacerbation of depressive and anxiety disorders [41-43].

The intricate interplay between obesity and mental health can be attributed to various factors, including biological mechanisms, societal stigmatization, and lifestyle factors [41]. Biological mechanisms involve the impact of adipose tissue on inflammatory processes and hormonal imbalances, contributing to changes in brain function and mood regulation. Societal stigmatization may result in increased stress and decreased self-esteem, further exacerbating mental health issues [38]. Additionally, lifestyle factors such as physical inactivity and poor dietary habits may contribute to both obesity and mental health disorders [39].

The implications of our findings extend beyond the academic realm, influencing public health strategies and interventions in Saudi Arabia. The significant associations between obesity and severe mental health outcomes underscore the need for integrated healthcare approaches that address both physical and mental well-being [43]. Public health interventions should prioritize early detection and management of mental health issues among individuals with higher BMIs [38].

Tailored interventions that consider cultural nuances and societal perceptions of obesity can enhance the effectiveness of mental health programs in Saudi Arabia. Addressing the stigma associated with obesity and fostering a supportive environment are crucial components of holistic healthcare strategies [42,43]. Collaboration between healthcare providers, policymakers, and community stakeholders is imperative to develop and implement comprehensive programs that address the complex interplay between obesity and mental health.

While our study provides insights, it is essential to acknowledge certain limitations. The cross-sectional nature of the included studies prevents the establishment of causal relationships. Additionally, variations in study populations, assessment tools, and methodologies may contribute to heterogeneity. Future research should prioritize longitudinal designs to elucidate the temporal dynamics of the obesity-mental health relationship and consider diverse population groups.

## Conclusions

Our meta-analysis contributes insights into the relationship between obesity and mental health in Saudi Arabia, which will subsequently contribute to the general knowledge of the subject. The significant associations observed with severe depression and anxiety emphasize the need for a holistic approach to healthcare that integrates mental health considerations into obesity management strategies. These findings have direct implications for public health policies, calling for the development of targeted interventions that address the complex interplay between physical and mental well-being in individuals with higher BMIs in Saudi Arabia.
